# You have to acknowledge the problem before you can address the problem: Audit looking at identification of co-existing substance misuse in a Liaison Psychiatry patient population

**DOI:** 10.1192/bjo.2021.276

**Published:** 2021-06-18

**Authors:** Emma McLean, Akash Kadiwar, Mariam Alexander

**Affiliations:** 1 Imperial College Healthcare NHS Trust; 2 Northampton General Hospital NHS Trust; 3 West London NHS Trust

## Abstract

**Aims:**

To evaluate if patients referred to Ealing Liaison Psychiatry Service (ELPS) with co-existing substance use are being appropriately identified as per NICE guidelines.

Patients with co-existing substance misuse have greater morbidity and mortality and it is therefore important to identify these patients to optimise their management. NICE recommends that all patients are asked about their substance use.

Anecdotally, our team felt we were doing a good job of identifying and managing such patients but we had no objective evidence of this.

**Method:**

Completed a retrospective audit looking at a sample of patients referred to ELPS over two weeks in December 2019.

A training session for ELPS was then held to highlight the initial audit results and NICE guideline recommendations.

We then repeated the audit over two weeks in March 2020.

**Result:**

Initial audit (100 patients):

Only 69% of patients asked about substance use. From those asked, 50-65.2% were using a substance, most commonly alcohol.

None of the patients over the age of 80 were asked about substance use vs 79.5% of patients aged 20–40 years.

55% of females vs 81% of males were asked about illicit substances.

33.3% of ward referrals vs 74.2% of Emergency Department referrals asked about substance use

Re-audit (53 patients):

Significant improvement across all areas

93% now asked about substance use

60% of over 80s, 96% of females and 85% of ward referrals were now being correctly asked about substance use

**Conclusion:**

We were surprised to find that we were initially not meeting NICE standards regarding asking patients about their substance use.

Acknowledging this problem during our training session proved to be effective.

This knowledge will help us develop our care pathways with our Acute colleagues and the Drug and Alcohol Liaison Service.

